# P-545. A Retrospective study of Respiratory Syncytial Virus Infection in Hospitalized Children Under Five Years with Lower Respiratory Tract Illness at a Tertiary Care Center in 2023

**DOI:** 10.1093/ofid/ofaf695.760

**Published:** 2026-01-11

**Authors:** Saugat Bhandari, Aayush Rizal

**Affiliations:** Patan Academy of Health Sciences, Lalitpur Kathmandu, Bagmati, 44700 Nepal, Bagmati, Nepal; Patan Academy of Health Sciences, Lalitpur Kathmandu, Bagmati, 44700 Nepal, Bagmati, Nepal

## Abstract

**Background:**

Respiratory Syncytial Virus (RSV) also known as human Respiratory syncytial viruses (HRSV) are well contagious respiratory pathogens & the leading cause of hospitalization due to Lower Respiratory Tract Infection (LRTI), especially in the pediatric population under 5 years. This study is intended to determine the RSV positivity in LRTI among < 5 year children, admitted at a tertiary care center in 2023.

**Methods:**

We retrospectively studied the cases with admission diagnosis of LRTI including all acute bronchiolitis from the file record section of the Patan Hospital, a tertiary care center. Both oropharnygeal and nasopharyngeal swabs were sent in all the cases. The swabs were sent to the National Public Health Laboratory were tested for RSV by real-time reverse transcriptase Polymerase Chain Reaction (RT-PCR) using CDC RSV Kit. Although the admissions were about 98, only 1/3rd of the cases were sent for PCV testing due to the limitation of testing in resource limited settings.

**Results:**

Among the 36 cases, 33% (12/36) were females and 67% (24/36) were males. The sex ratio was 2.0 among the all these cases. Out of the 36 cases sent for the RSV-PCR testing, 69.4% (25/36) were found to be positive for RSV. Out of the 12 female cases, 7 cases were positive among which 2 cases were RSV-B positive. Similarly, out of the total 24 male cases, 18 cases were positive among which 7 cases were RSV-B positive. Interestingly, the entire 36 cases positive for RSV had a median age of 3 years.

**Conclusion:**

The 69.4% RSV positive cases seen in the admitted LRTI children < 5 years is a sight of a concern. The observed predominance of RSV-B among cases further underscore the necessity for targeted interventions, including improved surveillance and vaccine development for children less than 5 years in resource limited settings in lower and middle incomes countries like Nepal. . Given the resource limitations in PCR testing, future studies should explore cost-effective diagnostic alternatives to ensure timely identification and management of RSV infections in vulnerable pediatric populations.

**Disclosures:**

All Authors: No reported disclosures

